# Social influences upon injection initiation among street-involved youth in Vancouver, Canada: a qualitative study

**DOI:** 10.1186/1747-597X-4-8

**Published:** 2009-04-30

**Authors:** Will Small, Danya Fast, Andrea Krusi, Evan Wood, Thomas Kerr

**Affiliations:** 1 British Columbia Centre for Excellence in HIV/AIDS, St. Paul's Hospital, 608-1081 Burrarrd St, Vancouver, BC V6Z 1Y6, Canada; 2 Department of Medicine, Faculty of Medicine, University of British Columbia, Vancouver, British Columbia, Canada

## Abstract

**Background:**

Street-involved youth are a population at risk of adopting injection as a route of administration, and preventing the transition to injection drug use among street youth represents a public health priority. In order to inform epidemiological research and prevention efforts, we conducted a qualitative study to investigate the initiation of injection drug use among street-involved youth in Vancouver, Canada.

**Methods:**

Qualitative interviews with street youth who inject drugs elicited descriptions of the adoption of injection as a route of administration. Interviewees were recruited from the At-Risk Youth Study (ARYS), a cohort of street-involved youth who use illicit drugs in Vancouver, Canada. Audio recorded interviews were transcribed verbatim and a thematic analysis was conducted.

**Results:**

26 youth aged 16 to 26 participated in this study, including 12 females. Among study participants the first injection episode frequently featured another drug user who facilitated the initiation of injecting. Youth narratives indicate that the transition into injecting is influenced by social interactions with drug using peers and evolving perceptions of injecting, and rejecting identification as an injector was important among youth who did not continue to inject. It appears that social conventions discouraging initiating young drug users into injection exist among established injectors, although this ethic is often ignored.

**Conclusion:**

The importance of social relationships with other drug users within the adoption of injection drug use highlights the potential of social interventions to prevent injection initiation. Additionally, developing strategies to engage current injectors who are likely to initiate youth into injection could also benefit prevention efforts.

## Background

The onset of drug injecting has been associated with elevated levels of health risks among young drug users [1]. New injectors often display high levels of syringe sharing [2], and HIV and hepatitis C (HCV) incidence is known to be elevated among young drug users who recently initiated injecting [1,3,4]. Street youth are one sub-population of drug users who are at heightened risk of adopting injection drug use [5,6], and the prevalence of injection drug use is known to be high among street youth in many urban settings [7-12]. Due to the pattern of injection-related harm evident among new injectors [13,14], the transition into injection drug use among non-injection drug users has become a focus for research and intervention efforts [15-17]. While further strategies to reduce injection-related harm among active injectors are urgently required [4], some have argued that preventing the initial adoption of injecting behaviour should be a greater priority for public health efforts [17,18].

The transition towards injection among young drug users is a complex process, influenced by an array of ecological factors [17]. Epidemiological studies have highlighted the role of social influences upon injection initiation, documenting that the majority of drug users are introduced to injection by an individual who is socially close to them [19-21], and in many instances new injectors are initiated by an injection drug user (IDU) who is several years older [22]. Gender differences in injection initiation experiences have been identified, as some studies have found that female drug users are more often introduced to injection by a male drug user [19,23,24], who is a boyfriend or sex partner. It appears that males often facilitate the injection initiation of female drug users; however, this is has not consistently been documented in all studies examining this topic [25]. Individual drug users also experience diverse trajectories in the transition surrounding injection initiation, including movements away from newly adopted modes of consumption which may be viewed as reverse transitions [26]. Although only a small number of qualitative studies have explored the transition towards injecting, these investigations have highlighted the importance of social circumstances and networks in facilitating the adoption of injection as a route of administration [27-29]. Both epidemiological research and intervention efforts could benefit from further qualitative accounts documenting the social processes surrounding the initiation of injection drug use [17], and contextualized understandings of the onset of injecting may help facilitate the development of appropriate interventions to prevent injection among street youth.

Vancouver, Canada, is a city with a large street youth population, characterized by high levels of drug use and homelessness [30,31]. Recent reports of high rates of crystal methamphetamine use [32] and overdose [33], resulting from both opiate and methamphetamine consumption, among local street youth indicate increasing public health problems within this population. Additionally, injection drug use is prevalent among local street youth, with 30% reporting recent injection drug use [7]. Alarmingly, high rates of syringe sharing are also evident with 29% of local street youth engaged in injection drug use reporting syringe sharing in the past six months [7].

In light of the emerging problem of injection drug use among street-involved youth locally, we undertook a qualitative study to explore the initiation of injection drug use among street youth in Vancouver. Our study is informed by the risk environment framework [34,35], which emphasizes the importance of ecological influences, including social and structural factors, in shaping patterns of drug-related risk and harm in a specific locale. More specifically, situated cultural norms, including beliefs regarding specific drug use practices (including routes of administration) and "folk" harm reduction strategies, may significantly shape the character of drug use behaviors among particular social groups of drug users [17,35]. Therefore, we focused this analysis upon social influences which shape the adoption of injection drug use among street youth in Vancouver.

## Methods

We conducted a series of 26 in-depth qualitative interviews to elicit accounts of the transition into injection, first injection experiences and uptake of injecting among young drug users. Interviews were conducted from September 2007 to January 2009, and interviewees were recruited from the At-Risk Youth Study (ARYS), a prospective cohort study composed of over 500 young street-involved drug users aged 16–26 in Vancouver [30]. First, in order to gather accounts from individuals who recently had initiated injection drug use, we identified and conducted interviews with 8 youth who had begun injecting over the previous 24 months. Secondly, in order to increase the overall number of qualitative study participants and permit consideration of a larger collection of accounts regarding injection initiation, we recruited an additional 18 study participants who had injected drugs prior to enrolling in the ARYS study. The second wave of individuals were selected from persons visiting the research office for cohort interviews, and during this round of recruitment, quotas were employed to ensure adequate representation of female and Aboriginal participants. These recruiting efforts created a sample of individuals who had experience injecting drugs, and which also reflected the socio-demographic profile of the larger cohort study. Interviews were undertaken by three trained interviewers (one male and two female) and facilitated through the use of an interview guide. Topics discussed included the transition towards injection, first injection experiences, ongoing injection drug use, and perceptions of injecting as a route of administration. Audio-recorded interviews lasted between 40 and 80 minutes and were transcribed verbatim.

The research team discussed the content of the interviews throughout the data collection process, thus informing the focus of subsequent interviews as well as the development of a coding scheme for partitioning the data categorically. The content of transcribed interviews was reviewed, and all text segments related to the adoption of injection drug use were catalogued. The catalogued data were subjected to a thematic analysis which focused upon the onset of injecting with attention to similarities and divergences in experiences. We present representative excerpts from the interview data to illustrate themes related to the uptake of injecting.

All participants provided informed consent to participate, and the study received ethical approval from the University of British Columbia Research Ethics Board. There were no refusals of the offer to participate in the interview and no drop-outs during interviews. All interviewees received CAD$20 for their participation.

## Results

The sample of 26 qualitative interview participants consisted of 12 females, 13 males, and one trans-gendered individual. The age of participants ranged from 16 to 26 years, and the median age of participants was 23. Table 1 depicts the demographics of the qualitative study sample compared to the larger group of young drug users participating in the ARYS study. Among the qualitative study sample the median age at first injection was 19, and among the entire ARYS cohort the median age at first injection in the entire cohort is 17 (IQR: 15–19).

**Table 1:** Characteristics of qualitative study sample compared to the composition of the At-Risk Youth Study (ARYS)

	**Qualitative Interview Participants**	**ARYS Cohort**
**Total Number**	26	529
**Median Age **(range)	23 (16–26)	21.9 (14.3–30.2)
**Gender**		
Female, n (%)	12 (46.2)	156(29)
Male, n (%)	13 (50.0)	371(70)
Trans-gendered, n (%)	1 (3.8)	2 (< 1)
**Aboriginal Ethnicity**		
Yes, n (%)	8(30.8)	127 (24)
No, n (%)	18(69.2)	879(81)

### The role of social influences in facilitating first injection experiences

According to participants, other drug users often played a significant role in instances when drugs were first injected. The majority of first injection experiences reported by participants involved an established injection drug user, who was known to the individual, introducing the initiate to injection drug use and facilitating initiation by providing guidance with the injection process. As indicated in the case of "G", a young man who was a heavy crack user and had previously smoked heroin, his closest friend was the person who introduced him to injecting:

*And it was raining, and then my best buddy...he comes along and he's like, you okay man? I'm like so, really choked, depressed. And he had a bunch of heroin on him. And he used to give me something to smoke too, right? But all this heroin was in one rig. He had a rig full... you know like it was a clean rig *[...]*So I guess it was just meant to be. And I was like, man I need some kind of a drug *[...]*"but, it's all liquid man." He had a shit load, he had all the supplies and everything like that. He did a little shot for me, and, as he was about to poke me right, and he looks at me he says, "God, strike me with lightening if you don't want me to do it.", but I was like " just do it, fuckin' hurry up." But I couldn't look, holy fuck- needles! *– Male Participant age 24, Interview #9

Similar to this description, several accounts of first injection experiences described a hesitancy to initiate injection among both the initiate and the individual initiating them.

Being injected by another individual was a common feature of first injection experiences among both male and female participants. Approximately half of the participants reported that their first injection was physically administered by the person who introduced them to injection as a mode of consumption. In some cases the initiator was the initiate's sex partner or boyfriend/girlfriend, and equal numbers of male and female participants reported that this dynamic characterized their first injection. In the instance of "D", a nineteen year old female drug user, her boyfriend was the person who facilitated her injection initiation:

*My ex-boyfriend, P, he... just happened to be a heroin addict. I mean I'd sit there watching him shoot up and stuff...he'd just do heroin in front of me and he'd just like keep hooking me up with jib *[methamphetamine], *and then I'd smoke some crack, and I was SO so out of my mind high by the third day. I hadn't slept... I told him, "I'm way too high ...can I do some heroin?" He was like, "No, no, no". And I was like, "Please. I need to like, settle down." ...that's the excuse, cause I'm not going to say, "Can I do some heroin? I've always wanted to do it." So then he's like, "Fine". And he was going to give me some tinfoil, to smoke it in. And I was like, "no, no, no, if I'm gonna do it, I want to shoot it". Like, that's the only way that I think heroin should be done*. [...] *And then he looked at my arm, "Fuck, you have awesome veins. I could hit them in the dark." yeah, and then he shot me up*. – Female Participant age 19, Interview #8

Although this participant was injected by her boyfriend, the account emphasizes that rather than being passive or coerced into experimenting with injection, female initiates may play an active role in their first injection experience. The account of a young woman who sought guidance with the injection process from an established injection drug user, who she knew through her social network, also illustrates how initiates may be active in bringing about their injection initiation:

*Well I knew that my boyfriend's mom had been doing it, for years and years. She's like 37 and, she's been using drugs for 20 years or something like that. I knew she knew how to inject it. And I didn't know anything about it, but...I had bought heroin, and I knew I wanted to try it for some reason. So, I asked her to do it for me. And, she did do it for me. [...] I wasn't really watching that closely but, I just remember she prepared it, 'cause she knew I didn't know how to do it*. – Female Participant age 22, Interview #1

Although a small number of participants' initiation did not involve another drug user who facilitated the first injection, these individuals had greater depth of previous exposure to injecting, with many reporting that members of their family were injectors who transmitted knowledge of injection techniques.

### Evolving perceptions of injection drug use and participation in injecting behaviour

Negative perceptions of injecting appear to be prevalent among street youth, and avoiding identification with "junkie" behaviour was a motivation for rejecting injection prior to initiation. Many participants reported that they did not anticipate they would ever adopt injection as a route of administration, and perceptions of injecting prior to initiation indicate that injection drug use represented a "risk boundary" [36]. An aversion to injecting behaviour and the use of syringes, as described in the following quote, was featured in many accounts detailing the transition towards injection:

*I didn't think I would ever do it. I was telling myself that I would never do it. Because I *[...] [didn't]*like needles before. And then I tried it*...-Male Participant age 20, Interview #18

While injecting was initially constructed as a "high-risk" and unacceptable behaviour among youth, accounts of the transition toward injecting indicate that negative perceptions were often replaced with the view that injection was an acceptable and expedient route of administration:

I: *What were your thoughts about injecting before that *[first injection]?

R: *I thought about trying it, and I thought that was a very bad idea, and it was disgusting... and then my thoughts turn to "hey, it's actually the cleanest and the fastest way to do it, so why not?" *– Female Participant age 22, Interview #11

The observation that injecting was more efficient than other routes of administration, which may enable a reduction in the amount of drugs used per dose, was a key dimension of the positive attributes ascribed to administration via injection:

*I got really high really fast, and you only need a small amount in order for you to get... super high*. – Female Participant age 16, Interview #13

Other participants also emphasized the importance of a more intense high as a key facet of the attraction of administration through injection.

The accounts of youth regarding initiation and subsequent patterns of injection emphasize the diversity of experiences, including the discontinuation of injection drug use after the first occasion and the existence of reverse transitions away from injecting. While positive perceptions of injecting were influential in the transition towards injecting, once the initial injection episode had occurred negative attributes of injection became more salient for some youth. A common belief among youth who continued injecting was that injection was a more "addictive" route of administration and was inherently more difficult to quit. The perception that continued injecting leads to increased dependency was also evident among youth who did not continue to inject, and was crucial in the motivation to avoid further injection drug use:

I: *So why'd you never inject again?*

R: *Because I noticed right away that I could become a junkie if I kept doing it. Cause morphine's pretty good. If you're having a hard day, you have a morphine pill*. – Male Participant age 22, Interview #10

Youth who did not establish a regular injection pattern, emphasized their rejection of "junkie" identity and the salience of negative perceptions of injecting within their social network:

R: *Heroin, no I'm going to stay away from that. Crack, I could fight that, I don't need it. I don't want to turn out like the people on Hastings. Man, I'm way better than that*.

I: *Thinking back to that time when you just tried that injection just once, what stopped you from injecting again?*

R: *Um, seeing junkies. And plus hearing about junkies, people disliking junkies and, like man, I don't want to be them*.-Male Participant age 23, Interview #4

Other participants who reduced their participation in injecting reported that strained social relationships and intervention on the part of their peers was an important factor in their trajectory away from injection:

I: *How did you stop injecting?*

R: *Um, my friends, really. My friends, 'cause they noticed, and I noticed, that I was going through some significant weight loss. I was starting to *[have]*borderline sociopath thoughts. I started thinking really insanely and violently and that kind of weirded people out. Yeah*, [friends]*they're like the slap in the head everybody kind of needs*. – Male Participant age 24, Interview #2

*I didn't want to get hooked on heroin, to the point where some people get really sick, so I stopped doing that. And then I stopped doing it with needles as well because other people that are close to me are shaking their heads at me because I use needles right? So then I was like, "whoa, I actually use needles". One thing that I said I'd never do, so I actually stopped*. – Female Participant age 22, Interview #17

Similar to the initiation of injection, the influence of other members of youth social networks and perceptions of injection drug use were central in transitions away from injecting. Notably, narratives describing transitions away from injecting were less evident in the accounts of female participants, and fewer female participants reported that they had ceased injecting.

### Social norms and sanctions regarding initiating others into injection

Reluctance on the part of the person initiating youth into injection was evident in many participants' first injection experiences, including the accounts presented above. Interview data revealed the existence of a "code" among local IDU which stipulates that young drug users should not be initiated into "hardcore" drug use, particularly injecting behaviour. The first injection experience of D, which was described above, illustrates how her boyfriend's initial reluctance to deliver an injection was related to the social convention which discourages initiating youth into injecting:

*And now if anyone asks him, he says that I forced him to*... [inject me] *I, you know, bribed him and fucking threatened him to do it. But really, as I remember it... he was convinced quite easily. And I don't hold it against him, right? But he used to get so defensive about it, 'cause ...he kinda had a little reputation for getting young girls that are new to downtown wired to heroin. He had nothing to do with me getting wired. I wasn't wired for a long time after I did that first one. But, it's funny 'cause, he totally didn't need any convincing, you know. I said, "I wanna' shoot", and he's like, "Ok, I'll doctor you"*. – Female Participant age 19, Interview #8

This account highlights that boyfriends and girlfriends are sometimes pressured to facilitate their partner's first injection, which may prevail over social norms discouraging initiating young users into injection. This interview also revealed that the boyfriend involved had a reputation as someone who had initiated multiple young females into injection drug use, and the significance of this code among local drug users is indicated by the fact that this perception precipitated difficulties for him within the street scene.

Participants who were established injectors described many situations where youth sought to inject and appealed to them to act as an injection guide. Some participants indicated that they attempt to discourage young users from adopting injection as a route of administration:

*'Cause even though I know how to do it and stuff, I don't like other people knowing- 'cause knowing how do to it is the main spark, right there*. – Male Participant age 24, Interview #2

These participants were adamant that they would avoid endorsing injection and refrain from initiating youth into injecting. Interview data suggests that the code discouraging initiating youth into injection is more prevalent among established injectors and those who have been injecting for a longer period of time, who likely have greater experience of negative consequences of injecting.

However, while many local drug users subscribe, at least superficially, to a code which discourages injection drug use among young drug users, our analysis indicates that this convention is routinely ignored. One participant who had sought help injecting from an older IDU, who was not well-known to her, provided an account which partially explains the motivations which may lead established injectors to deviate from this convention:

*When that girl injected me, I was fine and everything, I just didn't like the way it all happened. You know, she's the type of person that will go around saying, "Anyone who gives drugs to anybody under 19... fuck man, I'm gonna' punch them out." But then, because I had the money... She asked me how many times have I done it *[injecting], *and I said, "honestly, I've only done it once before, two days ago." And she's like, "Oh fuck, oh well." So, obviously because I'm paying for the drugs, who cares? She'll fucking inject me*.-Female Participant age 19, Interview #8

As indicated in this account, the ability to obtain drugs in exchange for assistance with the injection process may, for some established IDU, overwhelm concerns regarding initiating young people into injecting.

## Discussion

Our study is among the few qualitative studies examining the adoption of injection as a route of administration among street youth who use drugs, and our analysis underscores the importance of social influences upon the initiation of injecting. While initiates did play an active role in the initiation of injecting, the majority of first injection episodes involved another drug user facilitating the process, and this individual often physically administered the injection. We found that both the transition towards and movement away from injecting among new injectors is heavily influenced by the perceptions of peers and interactions with fellow drug users. These social interactions and perceptions of injecting serve to emphasize either the benefits or drawbacks of injection in comparison to other modes of consumption, and encourage or discourage injection drug use. It also appears that social conventions discouraging initiating young drug users into injection exist among many local IDU, although this code is often ignored.

Within our study, first injection episodes typically involved close friends, sex partners and older IDU known through social networks. These actors often facilitated the uptake of injecting, by creating a situation which presented an opportunity to inject within a familiar social context, and by providing injection expertise. Within our qualitative study sample, equal numbers of males and females reported being initiated by their sex partner or their boyfriend/girlfriend. However, epidemiological analysis of initiation patterns across the larger ARYS cohort indicate that females were more likely than males to report that their sex partner was involved in their injection initiation [37]. While romantic and sexual partnerships play an important role in structuring initiation experiences among women, this should not be interpreted to mean that women are inevitably coerced or persuaded into injection [38]. Our qualitative data indicates that female drug users often played an "active" role in bringing about their first injection experience, a dynamic which has been documented in other studies [25]. While previous qualitative research has emphasized that injection knowledge and skills are acquired from fellow drug users [29], within our study approximately half of the first injection episodes involved another drug user physically administering the injection. This is consistent with epidemiological studies documenting that first injection episodes often involve assisted injection [22,39]. It appears that this practice is particularly prevalent in our setting, as among a sample of 861 adult IDU in Vancouver, over 75% of first injections experiences involved assisted injections [40].

Some individuals in our study did not continue injecting after initiation, highlighting that the first injection experience does not immediately establish identification as an injector and that there is significant potential for transitions away from injecting among newly initiated youth [26,41]. Consistent with previous research, individuals who described these reverse transitions emphasized how negative perceptions of injection discourage injecting behaviour and peer intercession served to promote cessation [27], suggesting directions for future research and intervention efforts. Notably within our qualitative sample fewer female participants described reverse transitions away from injecting, and epidemiological examination across the entire ARYS cohort indicates that females were more likely to become regular injectors (injecting at least once a week) subsequent to their first injection, in comparison to males [37].

As young drug users are socialized into injecting [27,29], learning the behaviour and its meanings from other drug users, prevention efforts should adopt a social approach and develop peer interventions to complement conventional educational messages [1]. Participation in injecting is heavily influenced by socially constructed perceptions of injection [28], developed through social relationships and interactions with other drug users, rather than a rational calculation of the risks attached to injecting as a route of administration [36,42]. The perception that injecting resulted in a more intense high was one of the positive perceptions attributed to injecting within our study. In light of the fact that other studies have identified the "rush" associated with injecting to be an attractive feature of this route of administration [43,44], the role of pleasure in the adoption of injecting should receive greater consideration within prevention efforts. Our findings suggest prevention efforts targeting youth at-risk of injecting should deliver prevention messages through drug user networks in terms meaningful to youth [27,45].

Our qualitative study indicates that specific individuals may be involved in the initiation of multiple new injectors, and while this has been documented by epidemiological studies [46], future research should pay greater attention to the characteristics and motivations of IDU who initiate young drug users into injection, in order to develop intervention strategies which involve current injectors in prevention efforts [29]. This study is among the first to document a "code" among IDU in the Canadian context that discourages the initiation of young drug users into injection drug use. Although interview data revealed the existence of this social convention, it appears that many IDU are unable to adhere to this "moral code" due to the pressures of drug dependency and contextual factors which limit individual agency [47]. While current IDU do often initiate young drug users into injection, an ethic discouraging IDU from participating in the initiation of youth suggests the existence of "folk" harm reduction strategies, which could be incorporated into interventional efforts [48,49]. However, it may be that within injection-discordant couples, it is even more difficult for established injectors to refuse requests to initiate partners who currently use through non-injection routes, and these challenges should be prioritized within prevention efforts targeting current injectors. While prevention efforts targeting current IDU appear to be effective in reducing their involvement in the injection initiation of other users [16], interventions that reinforce negative perceptions of injecting may potentially exacerbate existing stigma and discrimination against established IDU [15,50,51].

The present study has a number of limitations. Although many interviewees had only very recently begun injecting, some participants may not have been able to recall details related to their injection initiation. However, previous research indicates that drug users possess adequate recall of early drug use experiences [52]. Additionally, as all participants offered retrospective narratives of early injection experiences, these may be subject to social desirability bias. While this analysis has concentrated upon social influences related to injection initiation, social processes and meanings among youth are also shaped by structural constraints and other contextual factors including poverty and homelessness. Lastly, our findings are based upon interviews with a relatively small sample of young IDU in Vancouver, and may not be representative of youth experiences with injection initiation in other urban settings.

In summary, our findings highlight the role social actors and processes play in the initiation of injection drug use, and further serve to underscore the potential of social interventions seeking to prevent initiation. Additionally, the reverse transitions away from injecting documented in this study suggest that interventions seeking to reduce the incidence of injection drug use among youth should also target new injectors.

## Competing interests

The authors declare that they have no competing interests.

## Authors' contributions

WS and TK designed the study. WS, DF and AK conducted all the qualitative interviews for this study, and performed the analysis of the interview data. WS prepared the first draft of the article. All authors commented on the original draft and contributed to the revision of the manuscript. All authors have approved the final manuscript.
